# A contribution to the Italian validation of the Parenting Interaction with Children: Checklist of Observations Linked to Outcome (PICCOLO)

**DOI:** 10.3389/fpsyg.2023.1105218

**Published:** 2023-03-29

**Authors:** Rosario Montirosso, Annalisa Castagna, Niccolò Butti, Mark S. Innocenti, Lori A. Roggman, Elisa Rosa

**Affiliations:** ^1^0-3 Center for the at-Risk Infant, Scientific Institute IRCCS “Eugenio Medea”, Lecco, Italy; ^2^Ph.D. Program in Neural and Cognitive Sciences, Department of Life Sciences, University of Trieste, Trieste, Italy; ^3^Institute for Disability Research, Policy, and Practice, Emma Eccles Jones College of Education and Human Services, Utah State University, Logan, UT, United States; ^4^Department of Human Development and Family Studies, Emma Eccles Jones College of Education and Human Services, Utah State University, Logan, UT, United States

**Keywords:** PICCOLO, Italian validation, mother-child interaction, parenting, child development

## Abstract

**Introduction:**

PICCOLO (Parenting Interactions with Children: Checklist of Observations Linked to Outcomes) is an observational instrument designed to assess four domains of parenting interactions that promote early child development (Affection, Responsiveness, Encouragement, and Teaching). Although PICCOLO has been validated in the United States for children as young as 4 months of age, the current focus is on parents with children aged between 10 and 47 months. This study contributes to the validation of the Italian translation of the PICCOLO by testing its psychometric properties and examining whether factors such as the child’s age and child’s sex are related to the four domains of parenting interactions.

**Methods:**

To these aims, 152 mothers of children aged 10–47 months from three Italian regions participated in the study.

**Results:**

Results indicate that the PICCOLO Italian version has acceptable inter-rater agreement, split-half reliability, and stability over time. Furthermore, the Italian version confirmed the robustness of the factor structure proposed in the original version. While there were no significant differences by child gender on the domains of parenting interactions, the Affection scores decreased with age.

**Discussion:**

Overall, these results demonstrate that the Italian version of the PICCOLO is a reliable measure of maternal interactions with children. The psychometric properties of the instrument make it appropriate for general research purposes and for assessment of parenting before and after support interventions.

## 1. Introduction

Parenting behaviors that support child development over time are referred to as *developmental parenting* (Roggman et al., 2008). Developmental parenting behaviors are crucial in promoting child development in both typically developing children, those with neurodevelopmental disabilities (i.e., psychomotor delay, cerebral palsy), and those at-risk for adverse outcomes, such as preterm children (Phillips and Shonkoff, 2000; Perrin et al., 2016; Festante et al., 2019). Different parenting domains are good predictors of positive developmental outcomes. Parents’ emotional warmth has been related to social emotional development and secure attachment (Laible et al., 2000; Zhou et al., 2002; Jeong et al., 2021). Responsive parenting promotes emotional, social, cognitive and linguistic development (Landry et al., 2006; Tamis-LeMonda et al., 2014; Jeong et al., 2021). Behaviors of encouragement, promotion of autonomy and cognitive stimulation (e.g., giving explanations and naming objects) have been associated with cognitive, linguistic and social development and school readiness (e.g., literacy skills) (Wade et al., 2018; Jeong et al., 2021).

Methodologically, it is important to be able to rely on instruments that can detect those specific parental behaviors that promote the emergence of the child’s competences. This could also help practitioners who work with parents assess parenting strengths and design tailored parenting support interventions. Several procedures have been suggested to examine parental functioning (Zaslow et al., 2006), but most of these instruments are limited to parent report, are psychometrically weak, or when they are based on direct observation of behavior, have time-consuming coding processes (Fuligni and Brooks-Gunn, 2013). We have not found a practical observational measure of parenting that has been empirically validated with Italian children. This limitation is especially concerning in clinical settings where it is essential to have valid and reliable tools to assess parents’ resources and strengths to plan parenting interventions and to monitor parenting outcomes (Aspland and Gardner, 2003; Zaslow et al., 2006). From a cross-cultural perspective, a tool that could be used among different countries and cultures is preferable (Bornstein et al., 1992).

In the light of these considerations Roggman et al. (2013a) developed PICCOLO (*Parenting Interactions with Children: Checklist of Observations Linked to Outcomes*), an observational tool which provides a parental functioning profile of parents of children between 10 and 47 months of age. The primary goal was the development of a measure that was useful to practitioners working with families. PICCOLO has been found useful for practitioners (Wheeler et al., 2013), has strong psychometric characteristics (Roggman et al., 2013b), and demonstrates strong predictive validity for both mothers and fathers (Anderson et al., 2013) and for children with and without disabilities (Innocenti et al., 2013). The PICCOLO is conceptually and empirically based on the theoretical perspective of developmental parenting (Roggman et al., 2013b), and has been translated into several languages including Spanish, German, Chinese, Turkish, Brazilian-Portuguese and Arabic. However, only three countries have validated the instrument on their population: Spain with a sample of 203 mother-infant dyads (Vilaseca et al., 2019a), Turkey with a sample of 156 dyads (Bayoglu et al., 2013) and Brazil-Portugal with 130 dyads (Schneider, 2018). The Italian adaption of PICCOLO can be useful to clinicians and researchers, especially given the lack of an observational instrument designed to assess the quality of parenting interactions which promote child development in Italy. The aims of present study were: (1) to contribute to the validation of the PICCOLO by testing its psychometric properties (reliability and factorial structure); and (2) to examine whether factors such as the children’s age and sex were related to developmental parenting.

## 2. Materials and methods

### 2.1. Participants

The Italian PICCOLO was administered to 152 mother-child dyads. The inclusion criteria were: (a) child’s age between 10 and 47 months; (b) full-term birth (after 37th week of gestation), natural childbirth and no complications in childbirth; and (c) absence of pre/post-natal pathologies and/or developmental difficulties as reported by parent. The sample was 55% male and 45% female, with 8% of the children under 1 year-old (10–11 months), 42% between 12 and 23 months, 28% between 24 and 35 months, and the remaining 22% between 36 and 47 months (M = 25.5, SD = 10.6). Mothers were between 23 and 50 years old (M = 36.4, SD = 4.0) and had high levels of education (M years of study = 17.2). The socio-economic family status (SES) was assessed using Hollingshead (1975) classification and the SES family scores were in the range from 10/100 (low socioeconomic level) and 90/100 (high socioeconomic level) (M = 68.9, SD = 16.0). Parents were informed about the general procedures and aims of the study and were asked to sign a written informed consent form. The study was approved by Ethics Committee of IRCCS “Eugenio Medea” and was conducted according to the World Medical Association’s Code of Ethics (World Medical, and Association., 2013).

### 2.2. Procedure

Recruitment was completed before pandemic restrictions took hold in March 2020 and was made possible thanks to the collaboration of pediatric centers and kindergartens located in three Italian regions: Lombardia, Emilia-Romagna and Marche. Researchers contacted kindergarten educator teams and pediatricians by letter or telephone and informed them of the study and its aims. In turn, educator teams and pediatricians invited parents who access their service to take part in the study. Parents who expressed interest in participating and who met the eligibility criteria were contacting by the study team. After determining the families’ availability to take part in the research, videotaping sessions were organized at either pediatricians’ offices or at kindergartens. The parents were informed that their participation would be entirely voluntary and anonymous. Information about the study, informed consent, and a demographic questionnaire were delivered to the parents prior the videotaping session. The observational setting consisted of a carpet measuring 2 m × 2 m in the middle of which was placed a box with some toys (rubber cubes, two small books, a puppet, a rattle, an electronic game); the camera was placed on a tripod at one side of the room and the researcher left the room leaving mother and child to interact during the recording. On the day of the appointment, after welcoming the dyads and familiarizing the child with the new environment and strangers, the recording began. Mothers were asked to interact with their son or daughter, for 10 min, according to the following instruction: “interact and play with your children as you typically do.” To assess test-retest reliability, a subsample of 20 dyads (average child’s age = 25.6 ± 9.5 months, nine males) was scheduled to take part in two observations (T1 and T2) carried out approximately 2 weeks apart in the same location.

### 2.3. PICCOLO

PICCOLO (Roggman et al., 2013b) is an observational tool designed to assess the quality of parenting interactions of parents of children aged between 10 and 47 months. The original version was developed and validated in USA on a large, diverse, primarily low-income sample (sample of over 2,000 families from different ethnic groups). PICCOLO has strong reliability and validity for typically developing children (Roggman et al., 2013b) and for children with atypical development or disabilities (Innocenti et al., 2013). PICCOLO scores from when children were in the first 3 years of life were found to predict improved academic outcomes for children when 11 years of age (Innocenti et al., 2013). PICCOLO scores were found to be even more predictive for children with atypical development or disabilities than for typically developing children. PICCOLO was reported by users to be an easy-to-use instrument. PICCOLO includes 29 items which are scored on a three-point rating scale, from 0 (absent, no behavior observed) to 1 (some brief or minor behavior) to 2 (clearly, strong or frequent behavior) based on a 10 min recording of parent-child free play interaction. The items are summed up into four domains that measure various aspects of parenting interactions that have been identified in the research literature as promoting early child development:

1. Affection (seven items) involves warmth, physical closeness and positive expressions toward the child (e.g., *shows emotional warmth)*;
2. Responsiveness (seven items) includes parental responses to child signals, emotions, words, interests, and behaviors (e.g., *pays attention to what the child is doing*);
3. Encouragement (seven items) includes active support for exploration, initiative, curiosity, creativity and play (e.g., *supports child in doing things on their own*);
4. Teaching (eight items) includes sharing play and interaction, cognitive stimulation, exploration and questions (e.g., *labels objects or actions for the child*).

The sum of the item scores contribute to a score for each domain between 0 and 14 (and 0 to 16 for Teaching domain) and to a total PICCOLO score.

### 2.4. Translation and adaptation

For the Italian translation of PICCOLO were used International Test Commission (ITC) guidelines for the translation and adaptation of tests (International Test Commission [ITC], 2017). First, following the ITC guidelines, permission was first obtained from the holder of intellectual property rights of the tool. The Italian version of PICCOLO was submitted to Brooks Publishing (Baltimore), which gave permission to proceed with the adaptation. Second, we used appropriate translation procedures to maximize the suitability of the tool adaptation in the Italian population. Here following the main steps performed to obtain the Italian PICCOLO: (1) translation from the original version (English) to the Italian; (2) an independent back-translation from the Italian to the English; and (3) developers (LR and MI) compared the two versions in the original language to determine to what extent both item versions capture the same parenting behaviors. When step 3 indicated a difference or differences between both item versions in the English, then the translation was revised to correct mistakes. Developers provided suggestions which were incorporated into the final Italian version. This process continued until no significant difference in the items meaning was found and the items version in the Italian was accepted. Translation team consisting of a professional translator and two psychologists with specific knowledge in the field of the developmental psychology and mother-child relationships developed the two first phases in the back-translation methods. The translation in Italian covered both the items and coding guidelines as well as the entire PICCOLO manual (Montirosso and Giusti, 2022) which was used for coders training and for coding the Italian sample. Third, we were careful to minimize the influence of potential cultural and linguistic differences that were relevant to the intended uses of the tool in the Italian context. For example, according with developers we have translated the name of first domain “Affection” with “Coinvolgimento emozionale” (“emotional involvement”). Indeed, the Italian term affection may have a slight different meaning than in English, as it also refers to an internal dimension (“to feel affectionate with my own child”) and it not only describes overt behaviors (“to exhibit affection at my own child”). Also cultural differences in parenting behaviors were taken into account by the authors of the Italian PICCOLO manual and further discussed during coders training.

### 2.5. Coder training

Three developmental psychologists working at the *0-3 Center for the at-Risk Infant* of the Scientific Institute IRCCS Medea coded the mother-child interaction videos according to the PICCOLO user’s guide (Roggman et al., 2013a). To reach a high level of reliability, the coders participated in a three-stage training process. First, the coders read the original manual and watched the PICCOLO™ training DVD (5 h). Next, each coder separately scored all videos provided on the DVD. Score differences between coders and the ratings reported in the original manual were discussed and consensus obtained. Lastly, coders scored eight videos of Italian mother-child interactions using the translated material (i.e., scoring sheets and guidelines) and discussed the differences in ratings until they came to an agreement for all items. The training was considered satisfactorily completed when the percentage of inter-rater agreement was equal to or above 80%. These three coders coded parenting behaviors in all video observations of parent–child interaction of the current study, which were used to analyze the psychometric properties of the Italian version of the tool. Each coder coded about one third of sample (Coder#1 = 56 videos; Coder#2 = 50 videos; Coder#3 = 46 videos).

### 2.6. Data analysis

First, descriptive statistics for each domain were calculated and Pearson’s correlations were conducted to verify that the PICCOLO domains met the recommended criterion for discriminant validity, which requires a correlation between two constructs of less than .85 (Kline, 1986).

Eighteen video observations (12%) were independently rated by two of the three coders and used to estimate inter-rater reliability, which reflects the variation between raters who measure the same group of subjects. Researchers have assessed inter-rater reliability with the intra-class correlation coefficient (Bartko, 1966), so here we used two-way random intra-class correlation coefficients (ICCs) at the domain levels. Additionally, we calculated the Cohen’s Kappa as measures of interrater concordance at the item levels.

Internal consistency was assessed through calculating Cronbach’s alpha coefficient for each domain and for the whole instrument.

In order to assess test-retest reliability, which is the stability in measurements taken by an instrument on the same subject under the same conditions, 18 dyads (children’s mean age = 25.11 months, SD = 9.76, 61% female) were recalled 2 weeks after the first observation (T1) and their interaction was again recorded and coded (T2). Student’s *t*-test for dependent samples was used to compare the domain scores at T1 and T2. Furthermore, to assess the presence of associations between T1 and T2, Pearson’s correlations between the scores obtained in the two observations were also calculated.

The validity of the Italian version of the PICCOLO was examined through confirmatory factor analysis, applying the factorial structure that emerged in the original version of the instrument in which the single dimensionality of each domain was tested. Goodness of fit of each model was assessed using the following criteria: relative chi-square (χ2/df): a good fit is indicated by a value lower than two; (b) comparative fit index (CFI) and Tucker-Lewis index (TLI): for both indices a value >90 indicates an acceptable fit; (c) root mean square error of approximation (RMSEA): a value of ≤0.08 RMSEA is indicative of an acceptable fit, while a value of ≤ 0.05 RMSEA is indicative of a good fit (Hu and Bentler, 1999).

The influence of child’s age and sex on the PICCOLO’s scores was then explored. In line with the Spanish validation (Vilaseca et al., 2019a), the sample was divided into three age groups: 12–23 months (M = 17.3, D.S. = 3.5); 24–35 months (M = 29.7, D.S. = 3.3); 36–47 months (M = 41.0, D.S. = 3.1). Preliminarily, differences between these age groups and between male and female participants in maternal variables (age, SES) were assessed through one-way Analysis of Variance (ANOVA) models and Student’s *t*-test, respectively. Thus, the scores obtained at the four PICCOLO domains were entered as dependent variables into a multivariate analysis of variance (MANOVA) with child’s age group and sex as between-subject factors. This approach was chosen since it combines multiple dependent variables into a single value, thus maximizing differences across groups, but also controls for the number of inter-correlations among them (Bray et al., 1985). Post-hoc comparisons were eventually carried out adopting the Bonferroni correction of the *p*-value.

Confirmatory factor analysis was performed using lavaan, an R package for Structural Equation Modeling, version 0.5–12 (Rosseel, 2012). The other statistical analyses were executed using IBM SPSS Statistics, version 22.0, with *p* ≤ 0.05.

## 3. Results

### 3.1. Correlational statistics for scores on the four PICCOLO domains

All Pearson coefficients between PICCOLO domain scores were statistically significant, with the only exception of the correlation between Affection and Teaching (*p* = 0.09). The lowest correlation coefficient was found between Responsiveness and Teaching, and the highest between Affection and both Responsiveness and Encouragement (see Table 1). Therefore, the PICCOLO domains were moderately correlated with one another, although not at a level that would suggest discriminant validity issues.

**TABLE 1:** Pearson’s correlation coefficients among Italian Parenting Interactions with Children: Checklist of Observations Linked to Outcomes (PICCOLO) domains (*N* = 152).

Domain	Affection	Responsiveness	Encouragement	Teaching
Affection	1.0	–	–	–
Responsiveness	0.40**	1.0	–	–
Encouragement	0.40**	0.38**	1.0	–
Teaching	0.09	0.20*	0.29**	1.0

*p < 0.05; **p < 0.01.

### 3.2. Interrater reliability

The ICCs were .98 for Affection, 0.99 for responsiveness, 0.97 for Encouragement, and 0.96 for Teaching, thus indicating an excellent reliability for all the domains. Similarly, Cohen’s Kappa values indicated a high inter-rater agreement at the item level within each domain (Affection: 0.82; Responsiveness: 0.83; Encouragement: 0.84; Teaching: 0.82).

### 3.3. Scale reliability

The alpha coefficient was satisfactory for the total score (0.68), while non-optimal values were reliable for the single domains (Affection: 0.45; Responsiveness: 0.53; Encouragement: 0.42; Teaching: 0.40).

### 3.4. Test-retest reliability

Test-retest reliability was based on observations at one time point (T1) compared with scores based on the observation of the same dyad 15 days later (T2). No difference emerged from *t*-test comparisons of domain scores between T1 and T2 (all *p* > 0.05), suggesting no statistically significant changes in PICCOLO scores over time. T1–T2 correlations were all significant (*p* < 0.05), with the exception of the Teaching domain (Affection: 0.83; Responsiveness: 0.55; Encouragement: 0.57; Teaching: 0.29), indicating that domain scores from the two observations were similar over the 2 weeks time period.

### 3.5. Confirmatory factor analysis

The results of the confirmatory factor analysis confirmed the one-dimensionality of the four domains, even though the saturation values were not optimal (0.29 for Affection, 0.39 for Responsiveness, 0.31 for Encouragement and 0.33 for Teaching). However, the model’s overall goodness of fit indices were acceptable: all chi-square values were not statistically significant and the association effect size indices were less than 2; all CFI and TLI values were greater than 0.90 and all RMSEA index values were less than 0.05.

### 3.6. PICCOLO scores by child’ sex and age

**FIGURE 1:**
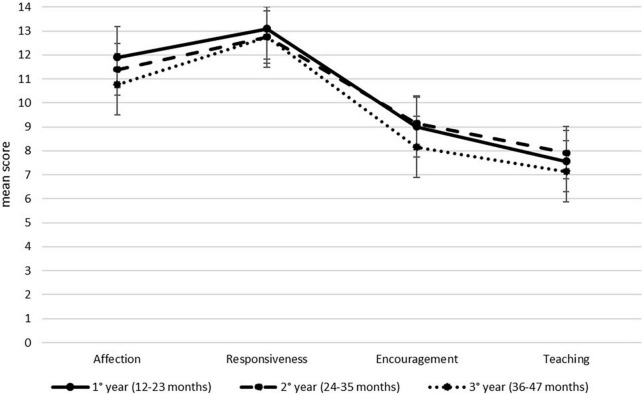
Parenting Interactions with Children: Checklist of Observations Linked to Outcomes (PICCOLO) domains in the three age groups.

Preliminary analyses did not reveal any statistically significant differences for age and gender (all *F* < 2.92, all *t* < 1.74, all *p* > 0.06) (Table 2). The MANOVA showed that neither age nor sex of the child, nor their interaction significantly affected the scores obtained across the four domains (all Wilks’s λ < 0.99, all *F* < 1.70, all *p* > 0.99). Nevertheless, between-subject effects revealed a significant influence of age group on the Affection domain (*F*_2,134_ = 4.42, *p* = 0.014, η^2^_*p*_ = 0.06). Post-hoc comparisons further clarified this effect showing that mothers of the younger group (12–23 months) obtained higher scores than mothers of the older group (36–47 months) (*p* = 0.008). All other between-groups effects were non-significant (*F* < 2.14, all *p* > 0.120). PICCOLO domain scores for the three age groups are showed in Figure 1.

**TABLE 2:** Infant and maternal demographic variables for the three age groups.

	Age group 1 (12–23 months)	Age group 2 (24–35 months)	Age group 3 (36–47 months)	Statistical comparison
*N* (m:f)	64 (37:27)	43 (20:23)	33 (22:11)	*χ2* = 3.18, *p* = 0.20
Mother’s age (year) Mean (SD)	36.8 (4.1)	35.5 (4.3)	37.2 (3.9)	*F* = 1.92, *p* = 0.15
Family SES Mean (SD)	68.3 (14.7)	73.6 (15.0)	65.1 (18.5)	*F* = 2.92, *p* = 0.06

## 4. Discussion

The primary aim of the present study was to determine if the Italian version of the PICCOLO was psychometrically valid. The inter-rater agreement, as assessed by ICC and Cohen’s Kappa, indicated a good reliability both at domain and item levels. As for the internal consistency, the Cronbach’s alpha coefficient was satisfactory for the total score and non-optimal for the single domains. Furthermore, test-retest reliability indicated a consistency of domain scores, suggesting good internal validity of Italian PICCOLO and that scores can be considered stable over time. The confirmatory factor analysis demonstrated the validity of the four-factors structure proposed in the original version (Roggman et al., 2013b). Testing the single dimensionality of each domain, the goodness-of-fit indices were at least acceptable, and better than those in the original version (RMSEA = 0.012) and in the Turkish validation (RMSEA = 0.11; CFI = 0.93) (Bayoglu et al., 2013), although in line with the Spanish validation (RMSEA = 0.057; CFI = 0.97) (Vilaseca et al., 2019a). Consistent with previous PICCOLO studies (Bayoglu et al., 2013; Roggman et al., 2013b,Vilaseca et al., 2019a), no differences in the domains scores emerged for child sex, while results indicated that mothers of younger children obtained higher scores in the Affection domain compared to mothers of older children. Altogether, the psychometric properties of the Italian PICCOLO confirm that the tool can be used as a valid instrument to identify strengths and measure progress in individual parents.

While reliability was overall good, internal consistency values of each domain were non-optimal. However, the excellent inter-rater reliability values reassure about the content validity of the PICCOLO and of each domain since, in an expert observation-based tool, higher interrater agreement should be found only when raters have a similar understanding of the assessed content (Krabbe, 2016). Moreover, it should be noted that automatic cut-off criteria may mislead the interpretation of the alpha coefficient, which should take into account the purpose of the instrument (Cho and Kim, 2015). The original developers of PICCOLO considered the purpose of the domain names and definitions not as labels for stable parenting traits, but instead as guidance for observers to identify behavior categories defined in the research literature using similar terms (e.g., warmth, sensitivity, support, stimulation) (Roggman et al., 2013b). The aim of PICCOLO was to identify observable parenting indicators, demonstrated by observer reliability, that would predict better developmental outcomes for young children, based on published research that linked the behaviors to positive developmental outcomes. Additionally, each of the final items predicted at least one child developmental outcome at age 3 years on measures collected concurrently with the parent-child observations (Early Head Start Research and Evaluation Project, Kisker et al., 1996). The authors’ goal was not to provide a measure of a unitary construct. Furthermore, the PICCOLO developers admit there are other positive parent-child behaviors in which parents may engage beyond those listed in the PICCOLO, but the goal of the PICCOLO was to identify a set of individually valid, reliable behavior items that were valid across all groups comprising the original sample of over 2,000 families (a larger sample than in any of the country validations). Consistent with an approach recommended by Bradley (2015), items were chosen because they represent phenomena and concepts (i.e., domains of parenting) that the literature indicated as important for child development, not because they were caused by a unitary underlying phenomenon. Certain items could be harder to see and lower in frequency, and thus less “internally consistent.” Nevertheless, all PICCOLO items predicted children’s development outcomes in the original measurement sample (Roggman et al., 2013a), thus being acceptable for an instrument assessing parenting behaviors within a developmental perspective.

The test-retest correlations suggested that the Teaching domain might be more susceptible to variation over time at the individual level. Contextual factors might have offered different opportunities for parents to express teaching behavior at T1 and T2, thus resulting in a different score distribution in the two sessions. However, no overall differences emerged when comparing the mean scores obtained in the two interactions, indicating that at a group level the teaching behaviors such as sharing play, providing cognitive stimulation, and labeling objects were stable across sessions. This confirms the PICCOLO as a valid instrument to assess parenting behavior over time. Individual differences in teaching behavior should be carefully considered.

In a similar vein, child’s age was found to affect scoring of the Affection domain, at least when comparing dyads with children aged 12–23 months with those aged 36–47 months. This finding is in line with previous validation studies of the PICCOLO in Turkey and Spain (Bayoglu et al., 2013; Vilaseca et al., 2019a), and is not surprising in a developmental perspective. Children older than 3 years old show indeed more exploration and autonomy behavior than younger children, thus influencing physical proximity to their parents, which is assessed by a specific item of the Affection domain. Future research should confirm this hypothesis and further explore how dyadic interpersonal distance could change during development and influence parenting behavior (Guida et al., 2021).

The multidimensionality of the parental profile assessed though the PICCOLO makes it possible to identify developmentally supportive parental behaviors on which to focus during parenting support interventions. Although this study did not include children with atypical development, these results encourage using the PICCOLO with parents of children at developmental risk or with neurodevelopmental disabilities, in keeping with the applications of the original version of this tool (Innocenti et al., 2013). Strengths identified by the PICCOLO can be shared with parents through feedback using either video or description. The methodology of video-feedback is recommended in order to provide a greater awareness of the relational dynamic between parent and child and provide useful indicators to support child development (Provenzi et al., 2020).

Results of this study should be read in the light of limitations. First, even though our results are similar to previous PICCOLO validation studies with comparable sample sizes in other countries (Bayoglu et al., 2013; Vilaseca et al., 2019a), our sample is small in comparison with the original PICCOLO measurement development sample. Furthermore, our parents were all mothers, therefore our results might not be generalizable to fathers (Anderson et al., 2013; Rivero et al., 2021). Future studies should validate PICCOLO in a wider Italian sample, considering gender differences in parental behaviors that have been well-established (Cabrera and Roggman, 2017). Please also note that this study was conducted in families mostly belonging to the middle and upper middle classes. Having a low income or limited education may certainly affect parenting behaviors (Roubinov and Boyce, 2017). Additionally, data were collected from only three Italian regions. Extending the sample to other areas would be important also in the light of possible cultural differences in parenting practice, that could indeed influence the PICCOLO scores. In this perspective, while the analyses confirmed the four-structure structure designed by the original authors, future research is needed to understand how cultural and contextual conditions might affect the parenting behaviors described by the PICCOLO across different countries (e.g., USA, Italy, and Spain) and different Italian regions. Moreover, the sample is not distributed proportionally across the three considered groups of age (10–11, 12–23, and 36–47 months) with a prevalence of children between 12 and 23 months. This could generate a bias in parenting behaviors that could be influenced by this age range. Overall, these limitations ask for caution when generalizing our findings to the whole population of Italian parents.

## 5. Conclusion

Despite these caveats, our findings suggest acceptable psychometric properties of the Italian PICCOLO. The Italian PICCOLO is a valid tool that can be used with Italian parents in clinical practice to detect and monitor parental functioning based on the four domains proposed by the authors in their developmental parenting model (Roggman et al., 2013b), and as a basis for implementing parenting support interventions (e.g., video feedback). Moreover, results of the present study suggest that the PICCOLO could be used in research context, as a possible outcome measure of parenting support intervention or as a proxy of parenting behaviors in conditions of typical and atypical development (Vilaseca et al., 2019b).

In sum, these results are encouraging for those who wish to use the PICCOLO in Italy for clinical and research purposes as a reliable, valid, and efficient instrument for detecting parental strengths and for helping parents to increase support for their children’s development. Accordingly, we emphasize the practical implications of PICCOLO for parenting intervention programs using a family centered approach (Giusti et al., 2018; Montirosso et al., 2020; Provenzi et al., 2020; Boudon et al., 2021). Further research should further confirm the validity of the Italian PICCOLO version through indices of external validity and assess its reliability and validity with broader samples, particularly to clinical samples such as at-risk children (i.e., preterm) and children with neurodevelopmental disabilities.

## Data availability statement

The raw data supporting the conclusions of this article will be made available by the authors, without undue reservation.

## Ethics statement

All procedures of this study were approved by Ethics Committee of IRCCS “Eugenio Medea”. Written informed consent to participate in this study was provided by the participants’ legal guardian/next of kin.

## Author contributions

RM: concept and design. NB, AC, and ER: study concept and design. NB and ER: acquisition of data. NB, AC, and RM: analysis and interpretation of data. RM, MI, and LR: drafting and critical revision of the manuscript for important intellectual content. All authors contributed to the article and approved the submitted version.
